# Characterization of an Amorphous Titanium Oxide Film Deposited onto a Nano-Textured Fluorination Surface

**DOI:** 10.3390/ma9060429

**Published:** 2016-05-31

**Authors:** Pei-Yu Li, Hua-Wen Liu, Tai-Hong Chen, Chun-Hao Chang, Yi-Shan Lu, Day-Shan Liu

**Affiliations:** 1 Institute of Electro-Optical and Materials Science, National Formosa University, Huwei, Yunlin 63201, Taiwan; 10476106@gm.nfu.edu.tw (P.-Y.L.); Neroliu0416@gmail.com (H.-W.L.); dream13130766@yahoo.com.tw (Y.-S.L.); 2 ITRI South, Industrial Technology Research Institute, Liujia Shiang, Tainan 73445, Taiwan; a0922639175@gmail.com (T.-H.C.); tsaicc1221@gmail.com (C.-H.C.)

**Keywords:** photocatalytic activity, two-step deposition, amorphous titanium oxide, nano-textured surface

## Abstract

The photocatalytic activity of an amorphous titanium oxide (a-TiO*_x_*) film was modified using a two-step deposition. The fluorinated base layer with a nano-textured surface prepared by a selective fluorination etching process acted as growth seeds in the subsequent a-TiO*_x_* deposition. A nanorod-like microstructure was achievable from the resulting a-TiO*_x_* film due to the self-assembled deposition. Compared to the a-TiO*_x_* film directly deposited onto the untreated base layer, the rate constant of this fluorinate-free a-TiO*_x_* film surface for decomposing methylene blue (MB) solution that was employed to assess the film’s photocatalytic activity was markedly increased from 0.0076 min^−1^ to 0.0267 min^−1^ as a mechanism for the marked increase in the specific surface area.

## 1. Introduction

Titanium oxide (TiO*_x_*) is one of the most popular materials in environmental purification, anti- or high-reflection coating, dye-sensitized solar cells, surface self-cleaning/antifogging functional coating, and biomedical engineering applications due to its chemical stability, optical transparency at visible wavelength, high refractive index, non-toxic nature, and low cost [1,2,3,4,5]. In terms of the photocatalytic activity, TiO*_x_* film is known to be influenced by its crystalline structure, specific surface area, and functional group incorporation [6,7]. Although the TiO*_x_* film with anatase structures has the best photocatalytic activity, the deposition and/or post-annealing temperature required to form anatase crystallinity is too high to limit such film applied to surface modification and packaging on the heat-sensitive substrates or devices. Accordingly, an effort was made to enhance the photocatalytic activity of an anatase-TiO*_x_* film at low temperature by realizing the *p*-*n* junction or doping with a specific dopant [8,9,10]. Alternatively, an amorphous TiO*_x_* film abundant in hydroxyl (O–H) functional groups on the film surface was found to be a promising substitute for exhibiting quality photocatalytic activity at a low temperature [11,12]. In our previous report, we developed a selectively fluorinated etching on the a-TiO*_x_* film to result in a nano-textured surface with the incorporation of fluorine ions [13]. Such film exhibited a high photocatalytic activity that was comparable to that of the TiO*_x_* film with an anatase structure and was applied to package the blue-light organic light emitting diode to result in a device with a self-cleaning function [14]. The apparent improvement on the film’s photocatalytic activity was a mechanism of the formation of the Ti–F functional group for facilitating the separation of the photo-produced charge couples and the increase in the reactants’ adsorption as a consequence of the large specific surface area. However, since the fluorine ion is not environment friendly for the most chemically reactive and electronegative of all the elements, it becomes increasingly necessary to prepare a fluorine-free surface of the a-TiO*_x_* film with the same photocatalytic activity by further optimizing the film’s specific surface area. Accordingly, the above-mentioned process should be improved to realize a quality a-TiO*_x_* film without the incorporation of the fluorine ions.

As the surface property of the a-TiO*_x_* film was modifiable by the selectively fluorinated etching, in this work, a base layer with such a nano-textured surface abundant in fluorine ions affecting the subsequently deposited a-TiO*_x_* film was studied. The growth mechanism of the resulting a-TiO*_x_* film with the fluorine-free surface prepared using this two-step deposition was elucidated through the observations of the associated surface and cross-section morphologies. The origin responsible for the improvement of the photocatalytic activity was characterized by an increase in the specific surface area and amounts of OH groups as conducted from the measurements of the surface roughness and chemical bond configuration.

## 2. Experimental Procedure

A 100-nm-thick hydro-generated a-TiO*_x_* film was deposited on silicon substrates (10 × 10 mm^2^) by a plasma-enhanced chemical vapor deposition (PECVD) system, using a titanium tetraisopropoxide [Ti(OC_3_H_7_)_4_, TTIP]-oxygen gas mixture. The deposition pressure, power, and gas flow rate of TTIP/O_2_ were controlled at 40 Pa, 100 W, and 120/20 sccm, respectively. A detailed system setup and deposition parameters have been described elsewhere [12]. Incorporating fluorine ions into the nano-textured surface on the a-TiO*_x_* film was achieved by pre-irradiating a UV light for 5 h through an anodic alumina membrane mask and then etching in the diluted hydrofluoric (HF) solution for a different time. The selective fluorination etching (hereafter abbreviated to SFE) has been addressed elsewhere [13]. A 100-nm-thick a-TiO*_x_* film using PECVD under the same deposition condition was then coated onto the base layer with surface nano-textures to realize the a-TiO*_x_* film surface free of the fluorine ions. In addition, another set of the a-TiO*_x_* film crystallized into anatase structures was prepared by post-annealing the un-treated a-TiO*_x_* film at 500 °C for 30 min under oxygen ambient (hereafter denoted as annealed TiO*_x_* film) as a comparison.

Film thickness of these a-TiO*_x_* films with and without the SFE treatment as well as prepared using the two-step deposition was measured using a surface profile system (Dektak 6M, Veeco, New York, NY, USA). Surface roughness was measured using atomic force microscopy (AFM, DI-3100, Veeco, New York, NY, USA) with the tapping mode. The surface and cross-section morphologies were observed by a field emission scanning electron microscope (FE-SEM, JSM-6700F, JEOL, Tokyo, Japan) operated at 3 kV. Fourier transform infrared spectrometry (FTIR, FT/IR-4100, JASCO, Halifax, NS, Canada) and X-ray photoelectron spectroscope (XPS, ULVAC-PHI, Quantera SXM, Kanagawa, Japan) with monochromatic Al *K*α radiation were employed to examine the film’s chemical bonding states and surface bond nature. The photocatalytic activity for the a-TiO*_x_* and annealed TiO*_x_* films illuminated by the UV light with a constant intensity of 1 mW/cm^2^ was evaluated by the decolorization of a 20 ppm concentrated methylene blue (MB) solution using the UV-Vis spectrophotometer from the absorbance of the resulting solution at 665 nm under atmosphere ambient.

## 3. Results and Discussion

**Figure 1:**
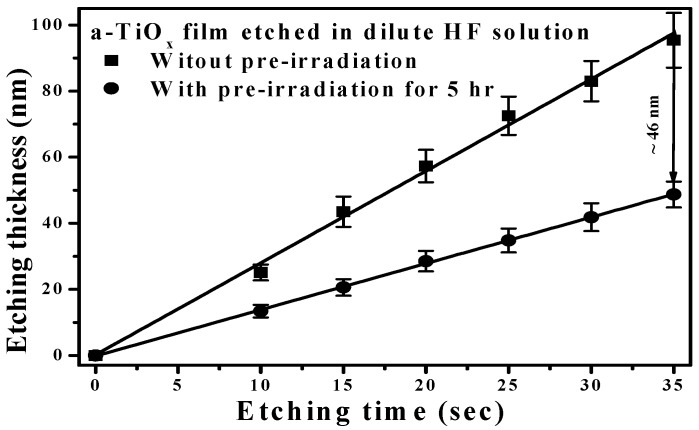
Etching thickness of the a-TiO*_x_* film with and without the UV light pre-irradiation and then immersed in the HF etching solution for different etching times.

Figure 1 shows the etching thickness of the a-TiO*_x_* film with and without the UV light pre-irradiation for 5 h, and then immersed in the HF etching solution for different etching times. The etching thickness of the a-TiO*_x_* film treated by additive UV light pre-irradiation was apparently lower than the a-TiO*_x_* film directly etched by the HF solution. The less acidic surface of the a-TiO*_x_* film, as a consequence of the Ti(IV)−OH, transformed into a Ti(III)−OH^−^ group due to the generated electrons under UV light pre-irradiation, was responsible for the alleviation of the sequential etching process [13]. A large discrepancy occurred in the etching thickness between the a-TiO*_x_* films with and without the UV light pre-irradiation (approximately 46 nm) as the etching time reached 35 s, while at this time the 100-nm-thick a-TiO*_x_* film directly etched by the HF solution was almost completely removed, as shown in Figure 1. Because an apparent difference in the etching thickness of the a-TiO*_x_* films was obtainable from the fluorination etching with and without UV light pre-irradiation, a selective fluorination etching treatment on the a-TiO*_x_* film to modify its surface morphology was carried out by selectively shadowing the surface through a nano-sized mask with UV light pre-irradiation and then processing the fluorination etching.

**Figure 2:**
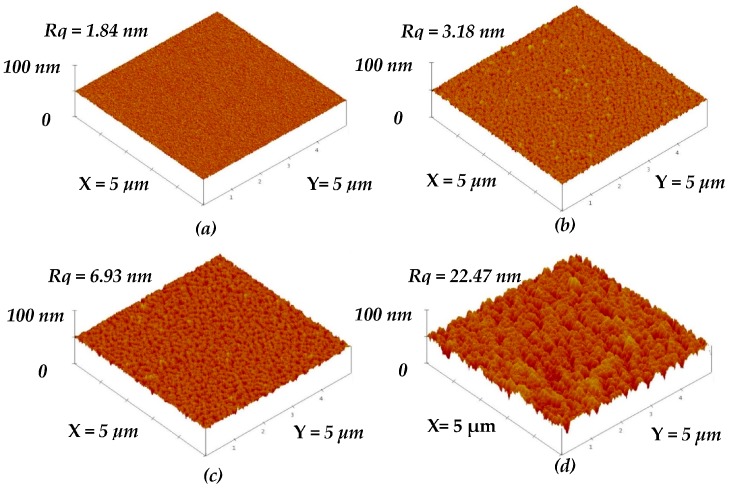
Surface roughness of the (**a**) un-treated a-TiO*_x_* film; and SFE-treated a-TiO*_x_* film for (**b**) 10; (**c**) 20; and (**d**) 35 s; respectively.

Figure 2b–d show the surface roughness of the a-TiO*_x_* film selectively etched by the HF solution for 10, 20, and 35 s, respectively (the un-treated a-TiO*_x_* film is given in Figure 2a for comparison). The untreated a-TiO*_x_* film exhibited a smooth surface with a roughness of about 1.84 nm, whereas an increase in the surface roughness was obtained from the a-TiO*_x_* films treated by the additive SFE process. The voids on the a-TiO*_x_* surface gradually became visible as the SFE-treated time increased. Notable pinnacles and valleys were then clearly observed from the surface of the a-TiO*_x_* film treated by the SFE process for 35 s, which corresponded to a very high surface roughness of 22.47 nm.

**Figure 3:**
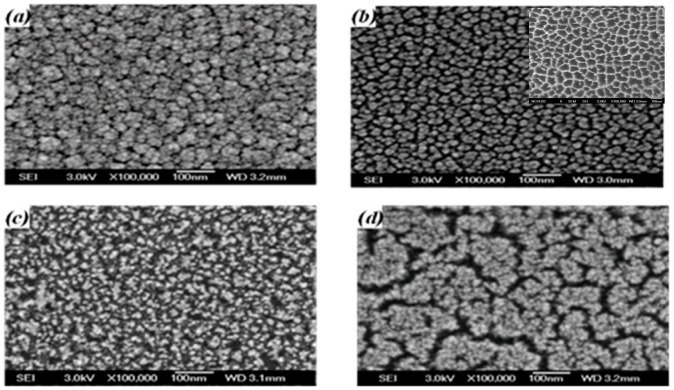
Surface morphologies of the (**a**) untreated a-TiO*_x_* film; and SFE-treated a-TiO*_x_* film for (**b**) 10; (**c**) 20; and (**d**) 35 s, respectively (the AAM patterns is provided in inset figure of Figure 3b).

The surface morphologies of the untreated a-TiO*_x_* film and the films treated by the SFE process for 10, 20, and 35 s, respectively, are shown in Figure 3a–d. As shown in Figure 3a, the particles on the untreated a-TiO*_x_* surface are distributed densely and abnormally, whereas these particles observed from the a-TiO*_x_* film surface processed by the SFE treatment for 10 s became separated with a round-like shape. The SFE process also resulted in these particles being uniformly distributed with an average diameter of about 20 nm, which was similar to the porous size of the AAM mask, as shown in the inset figure. Fine particles were then observed from the a-TiO*_x_* film surface as it was further treated by the SFE process for 20 s (Figure 3b). The sidewall etching that led to the overcut profile was one possible reason why these particles appeared on the surface, evolving from a round-like to a needle-like shape. Meanwhile, the corresponding surface roughness of the a-TiO*_x_* film also increased from 3.18 to 6.93 nm, as measured from Figure 2c. When the SFE treatment on the a-TiO*_x_* film reached 35 s, the surface morphology also showed definite boundaries in addition to the fine particles. Referring to the etching thickness of the a-TiO*_x_* film given in Figure 1, the obvious and wide boundaries were attributed to the regions of the a-TiO*_x_* film that was completely removed from the substrate after etching by the HF solution without additive UV light irradiation.

**Figure 4:**
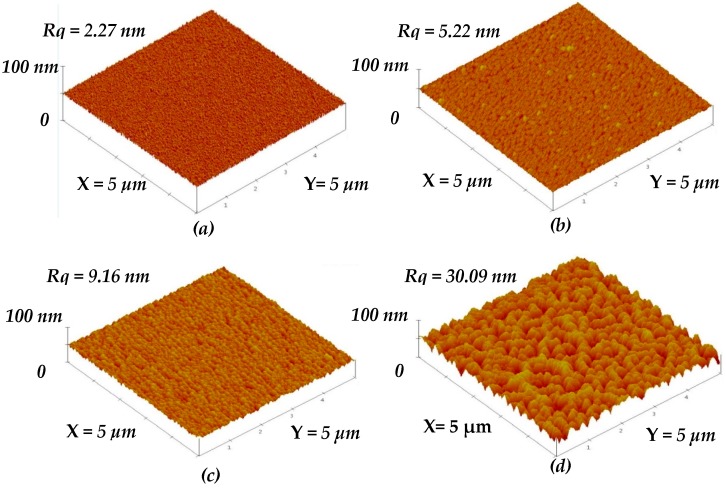
Surface roughness of the a-TiO*_x_* film deposited onto (**a**) untreated base layer; and SFE-treated base layers for (**b**) 10; (**c**) 20; and (**d**) 35 s; respectively.

These experiments demonstrated that the particles’ shape and distribution on the a-TiO*_x_* film surface were modifiable and controllable using the SFE treatment for different times. These base layers with surface nano-textures affecting the subsequent deposited a-TiO*_x_* film were then investigated. Figure 4b–d give the surface roughness of the a-TiO*_x_* films deposited onto the SFE-treated surface with nano-textures shown in Figure 2b–d, respectively, as well as the film deposited onto the untreated surface (Figure 4a). All the a-TiO*_x_* films prepared using this two-step deposition process exhibited a higher surface roughness than that of their base layers shown in Figure 2a–d. The surface roughness of the a-TiO*_x_* film deposited onto the untreated surface increased slightly to 2.27 nm. In contrast, a marked increase in the surface roughness was measured from the a-TiO*_x_* film deposited onto the SFE-treated surface. The rougher the surface of the base layer obtained, the higher the increase in the roughness of the subsequently deposited a-TiO*_x_* film. In addition, features of the pinnacles and valleys appearing on the surface of the a-TiO*_x_* film deposited onto the surface treated with SFE for 35 s (Figure 4d) became more visible compared to those observed from the surface of its base layer (Figure 2d).

**Figure 5:**
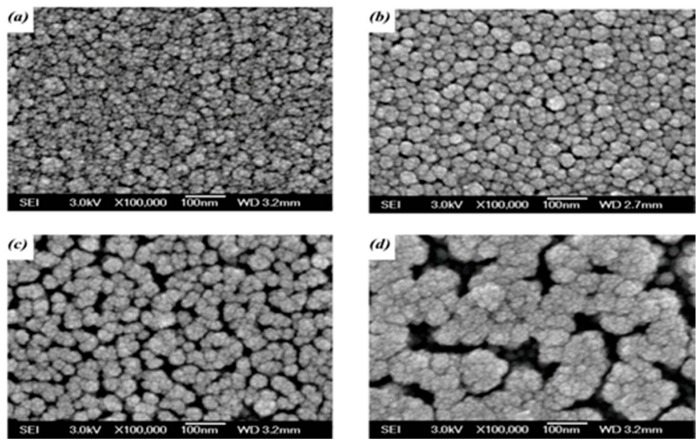
Surface morphologies of the a-TiO*_x_* film deposited onto (**a**) untreated base layer; and SFE-treated base layers for (**b**) 10; (**c**) 20; and (**d**) 35 s; respectively.

The corresponding surface morphologies shown in Figure 4 conducted from SEM measurements are presented in Figure 5a–d. The surface morphology of the a-TiO*_x_* film deposited onto the untreated surface (Figure 5a) was almost identical to the film deposited onto the substrate on which the particles were distributed randomly and densely as presented in Figure 3a. For the a-TiO*_x_* film deposited onto the surface with round-like particles, as shown in Figure 3b, the particles appearing on the a-TiO*_x_* film (Figure 5b) had an average diameter apparently larger than those distributed over the base layer’s surface, although their shape and distribution were quite similar. This suggested that the nuclei of the subsequently deposited a-TiO*_x_* film were prone to forming and growing along the particles distributed over the base layer. The growth of particles on the a-TiO*_x_* film became closer and resulted in the considerable increase in the surface roughness. For the a-TiO*_x_* film deposited onto the surface shown in Figure 3c, the nuclei growing along the needle-like structure resulted in the particles on the a-TiO*_x_* film surface evolving into a round-like shape with visible boundaries. As the surface morphologies of the two-step a-TiO*_x_* films were deeply relevant to the particles on the base layer, the distributions of the significant channels and clusters observed from the a-TiO*_x_* film shown in Figure 5d consisted of the growth of the fine particles and the enhancement of the boundaries appearing on the base layer shown in Figure 3d.

**Figure 6:**
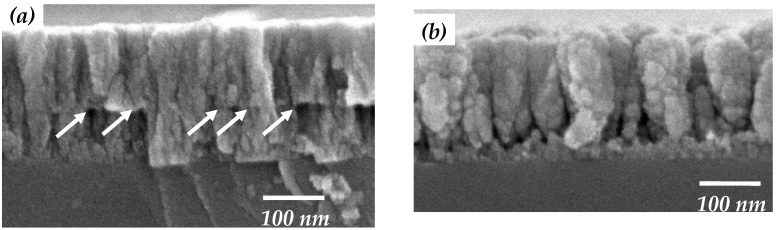
Cross-section images of the a-TiO*_x_* film deposited onto (**a**) untreated; and (**b**) SFE-treated base layers (the arrows in Figure 6a mark the interface of the two-step deposited a-TiO*_x_* film).

The cross-section micrographs shown in Figure 6a,b give further insights into the growth of the a-TiO*_x_* film prepared using the two-step deposition process. In Figure 6a, random and dense fiber-like structures can be seen in both a-TiO*_x_* layers, which had a definite interface, as indicated by arrows. This implied that the untreated base layer would not cause a structural change in the subsequently deposited a-TiO*_x_* film. In contrast, the cross-section of the two-step a-TiO*_x_* film shown in Figure 6b exhibited the feature of nanorod-like structures with no significant interface, confirming that the growth mechanism of the a-TiO*_x_* film deposited onto the SFE-treated base layer was completely different from the film deposited onto the untreated base layer. The dimension of these nanorod-like structures was found to be widened with the growth of the two-step a-TiO*_x_* film. Combined with their surface morphologies (Figure 3d and Figure 5d), the particles on the base layer achieved using the SFE treatment were likely to act as growth seeds for the subsequent a-TiO*_x_* deposition, thereby resulting in the upper a-TiO*_x_* film, demonstrating conformity in the base layer with the increase in the surface roughness.

**Figure 7:**
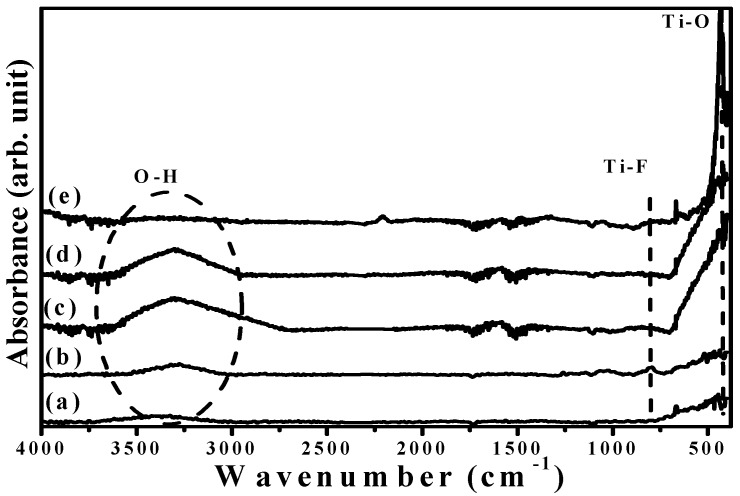
FTIR spectra of the (**a**) untreated; and (**b**) SFE-treated base layers and the a-TiO*_x_* films deposited onto the (**c**) untreated; and (**d**) SFE-treated base layers; as well as (**e**) the annealed TiO*_x_* film.

FTIR spectra of the base layer with and without an additive SFE treatment and the a-TiO*_x_* films prepared using two-step deposition are illustrated in Figure 7a–d, respectively (Figure 7e) also shows the FTIR spectrum of the annealed TiO*_x_* film for comparison). Both the untreated and SFE-treated base layers, as shown in Figure 7a,b, respectively, consisted of the fingerprint peak of the Ti–O bond around 400–800 cm^−1^ with the functional group of hydroxyl (OH) around high wavenumbers (2800–3700 cm^−1^) [12]. The SFE treatment also caused the base layer to emit an additive signal at about 820 cm^−1^, which was associated with the Ti–F vibration mode [13]. In addition, the incorporation of the fluorine ions into the base layer led to a shift of the O-H bond from 3450 to 3275 cm^−1^ due to an increase in the surface acidity. When the a-TiO*_x_* film was deposited onto the SFE-treated base layer, the Ti–F bond was hardly observed in the FTIR spectrum (Figure 7d). Compared with the FTIR spectrum of the a-TiO*_x_* film deposited onto the untreated base layer (curve c), both spectra only featured Ti–O and O–H bonds with almost the same peak position, except for a higher relative O-H bond intensity obtained from the a-TiO*_x_* film deposited onto the SFE-treated base layer. Since the porous structures distributed in the low-temperature deposited oxidation film were the main reason responsible for the formation of the O–H groups [15,16], the reinforcement in the O–H bond obtained from the two-step deposition of the a-TiO*_x_* coated onto the SFE-treated base layer thus implied the increase in the amounts of the pores. In addition, a sharp and intense Ti–O bond with the disappearance of the O–H bond as a consequence of the anatase crystallization was obtained from the FTIR spectrum of the annealed TiO*_x_* film.

**Figure 8:**
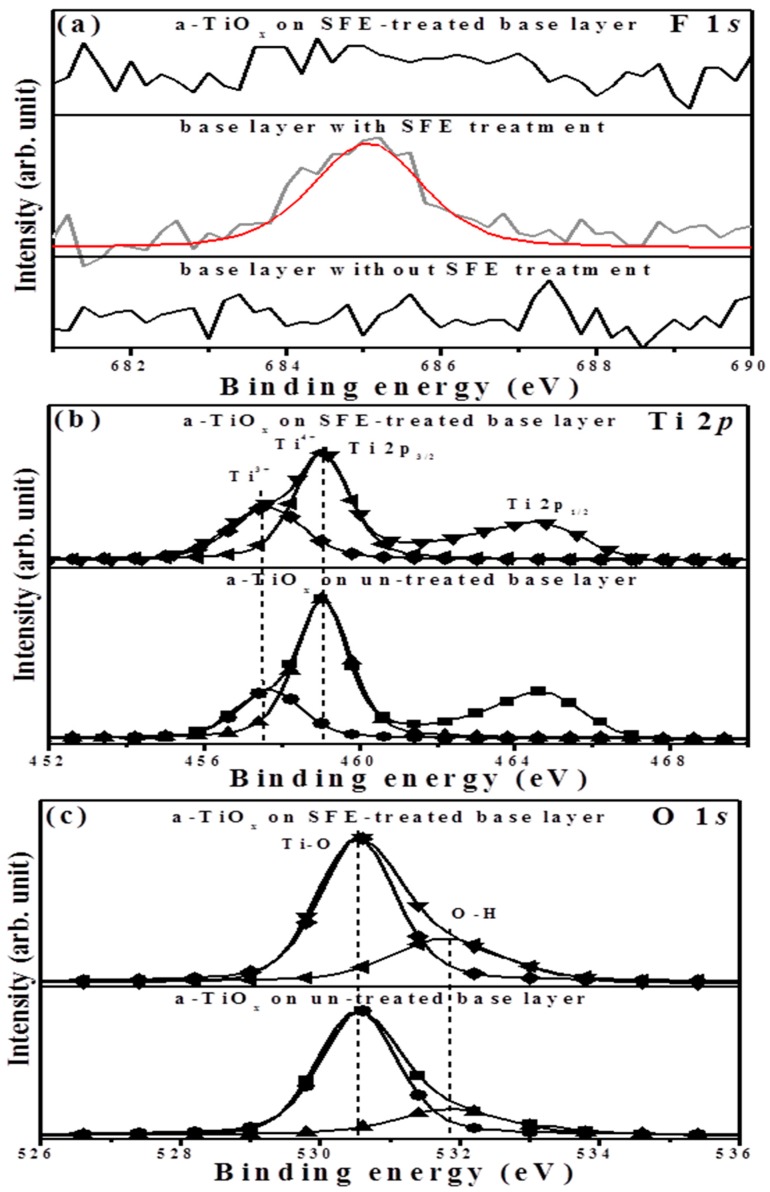
Binding energies related to the (**a**) F 1*s* core of the untreated and SFE-treated base layers as well as that of the a-TiO*_x_* deposited onto the SFE-treated based layer; (**b**) Ti 2*p*; and (**c**) O 1*s* core levels of the a-TiO*_x_* film deposited onto the untreated and SFE-treated base layers.

The binding energies related to the F 1*s*, Ti 2*p*, and O 1*s* core levels measured from the surface of the two-step deposited a-TiO*_x_* film as well as the base layer are respectively illustrated in Figure 8a–c. Although a significant fluorine signal on the surface of the base layer emerged at about 684.9 eV, which was assigned to the Ti–F chemical bond due to the SFE treatment [17], this signal was absent in the surface of the subsequently deposited a-TiO*_x_* film, indicating the achievement of the fluorine-free surface. In the Ti 2*p* spectrum (Figure 8b), both the peaks of the binding energies for the Ti 2*p*_1/2_ and Ti 2*p*_3/2_ occurred at approximately 464.6 and 458.9 eV, respectively, with a binding energy difference of 5.7 eV. A broad signal with a distinct satellite peak was observed from the binding energy of Ti 2*p*_3/2_ for the a-TiO*_x_* film deposited onto the SFE-treated base layer, while this peak became sharp with a tail extending to the low binding energy in the spectrum of the a-TiO*_x_* film deposited onto the untreated base layer. The peak of Ti 2*p*_3/2_ could be deconvoluted into two species of Ti^4+^ and Ti^3+^ ion states at 457.6 and 459.0 eV, respectively [18]. The composition of the Ti^3+^ to Ti^4+^ state (in the area of Ti^3+^/(Ti^4+^ + Ti^3+^)), which was associated with the deficiency in the oxygen atoms on the surface, apparently increased from 0.29 to 0.39 as the a-TiO*_x_* film was deposited onto the SFE-treated base layer. Regarding the O 1*s* spectra (Figure 8c), both surfaces of these two samples displayed an intense peak with a long tail extending to a high binding energy that could be deconvoluted into two feature peaks. The binding energy peak located at 530.6 eV was related to the Ti–O chemical bond, where the peak at the high binding energy of 531.8 eV emerged from the hydroxyl group (O–H) [19,20]. As determined from previous papers [21,22], the presence of the O-H bond indicated the termination of the chemical bond and/or contaminants and, thus, was responsible for the structural voids and boundaries. Accordingly, marked boundaries and voids observed from the surface and cross-section morphologies of the a-TiO*_x_* film deposited onto the SFE-treated base layer were hydroxylated more intensely in the binding energy of O 1*s* (approximately 0.31, in the area of O–H/(Ti–O + O–H)) than those of the film deposited onto the untreated base layer (approximately 0.24).

**Figure 9:**
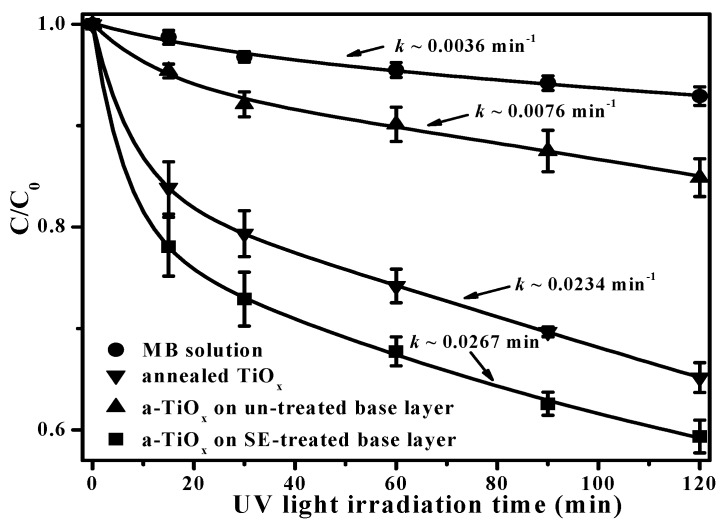
Photo-degradation to MB solution using a-TiO*_x_* films deposited onto untreated and SFE-treated base layers as well as using the annealed TiO*_x_* film (MB solution decomposed by the UV light irradiation also is shown for comparison).

In addition, because the hydroxyl groups are beneficial for trapping hole carriers to suppress the recombination of the photo-generated electron-hole pairs, as demonstrated in previous studies [23,24], the a-TiO*_x_* film deposited onto the SFE-treated base layer to degrade the MB solution, as depicted in Figure 9, is greatly improved compared to the same film deposited onto the untreated base layer. It is also worth noting that the a-TiO*_x_* film with a surface free of the fluorine ions achieved using the two-step deposition even exhibited better efficiency in decomposing the MB solution than the annealed TiO*_x_* film with anatase structures. The rate constant, *k*, which represents the quality of the photocatalytic activity of the film can be evaluated by fitting the curves shown in Figure 9, using the following relationship [25]:

(1) $$\ln\left(C/C_{0}\right)=kt$$

where *C* and *C*_0_ are the concentrations of the MB solution at a UV light irradiation time of *t* = 0 and *t*, respectively. The *k* value evaluated from each curve is denoted in Figure 9. Clearly, the a-TiO*_x_* film deposited onto the SFE-treated base layer corresponded to a rate constant of 0.0267 min^−1^, which was three times higher than the film deposited onto the untreated base layer (0.0076 min^−1^) as well as a little higher than that of the annealed TiO*_x_* film (0.0234 min^−1^). According to the investigations into the morphologies and the analysis of the chemical bond configurations of the two-step deposited a-TiO*_x_* film, the apparent roughening and nano-textured surface of the upper a-TiO*_x_* film without the incorporation of the fluorine ions that grew by conforming along the particle seeds on the base layer surface, which was achieved using an additive SFE treatment, was the mechanism responsible for the great enhancement in the resulting photocatalytic activity.

## 4. Conclusions

The depth-dependent morphology of the surface-fluorinated a-TiO*_x_* film was realized using an additive SFE treatment for different times. At the initial stage, the size and shape of these particles distributed over the a-TiO*_x_* film surface after treatment with the SFE process for 10 s conformed to the patterns of the shadow mask. As the SFE treatment increased to 20 s, the shape of the particles appearing on the a-TiO*_x_* film surface evolved from a round-like to a needle-like shape as a consequence of the apparent sidewall etching effect. Eventually, significant channels that corresponded to the film being completely removed from the substrate were observed from the a-TiO*_x_* film processed by an additive SFE treatment for 35 s. The fluorinated surface with specific nano-textures of the a-TiO*_x_* film acted as seed layer for the subsequently deposited a-TiO*_x_* film according to the investigations of the surface and cross-section morphologies of the resulting a-TiO*_x_* film. The nuclei of the a-TiO*_x_* film self-assembled on the particles distributed over the SFE-treated base layer and then grew up to cause an apparent increase in surface roughness. Such roughening of the surface without the fluorine ion incorporation was achieved using the two-step deposition due to the deposition being selective, which also resulted in the film surface being abundant in the hydroxyl groups that were helpful for suppressing the recombination of the photo-generated electron-hole pairs. Accordingly, the fluorine-free a-TiO*_x_* film deposited onto the SFE-treated base layer which had a nanorod-like structure possessing efficient photocatalytic activity, with a rate constant of 0.0267 min^−1^; this was much higher than that of the film deposited on the untreated base layer (~0.0076 min^−1^ ), as evaluated from these films photo-degrading in the MB solution.
